# A Statewide Analysis of the Incidence and Outcomes of Acute Mesenteric Ischemia in Maryland from 2009 to 2013

**DOI:** 10.3389/fsurg.2016.00022

**Published:** 2016-04-14

**Authors:** Robert S. Crawford, Donald G. Harris, Elena N. Klyushnenkova, Ronald B. Tesoriero, Joseph Rabin, Hegang Chen, Jose J. Diaz

**Affiliations:** ^1^Division of Vascular Surgery, Center for Aortic Disease, Department of Surgery, University of Maryland School of Medicine, Baltimore, MD, USA; ^2^Department of Epidemiology and Public Health, University of Maryland School of Medicine, Baltimore, MD, USA; ^3^Division of Acute Care Surgery, R Adams Cowley Shock Trauma Center, University of Maryland School of Medicine, Baltimore, MD, USA

**Keywords:** acute mesenteric ischemia, epidemiology, surgical critical care, emergency surgery

## Abstract

**Introduction:**

Acute mesenteric ischemia is a surgical emergency that entails complex, multi-modal management, but its epidemiology and outcomes remain poorly defined. The aim of this study was to perform a population analysis of the contemporary incidence and outcomes of mesenteric ischemia.

**Methods:**

This was a retrospective analysis of acute mesenteric ischemia in the state of Maryland during 2009–2013 using a comprehensive statewide hospital admission database. Demographics, illness severity, comorbidities, and outcomes were studied. The primary outcome was inpatient mortality. Survivors and non-survivors were compared using univariate analyses, and multivariable logistic regression analysis was performed to evaluate risk factors for mortality.

**Results:**

During the 5-year study period, there were 3,157,499 adult hospital admissions in Maryland. A total of 2,255 patients (0.07%) had acute mesenteric ischemia, yielding an annual admission rate of 10/100,000. Increasing age, hypercoagulability, cardiac dysrhythmia, renal insufficiency, increasing illness severity, and tertiary hospital admission were associated with development of mesenteric ischemia. Inpatient mortality was high (24%). After multivariate analysis, independent risk factors for death were age >65 years, critical illness severity, mechanical ventilation, tertiary hospital admission, hypercoagulability, renal insufficiency, and dysrhythmia.

**Conclusion:**

Acute mesenteric ischemia occurs in approximately 1/1,000 admissions in Maryland. Patients with mesenteric ischemia have significant illness severity, substantial rates of organ dysfunction, and high mortality. Patients with chronic comorbidities and acute organ dysfunction are at increased risk of death, and recognition of these risk factors may enable prevention or earlier control of mesenteric ischemia in high-risk patients.

## Introduction

Acute mesenteric ischemia is the final common pathway of a range of conditions that lead to critical malperfusion of the small and/or large intestine (1–3). Mesenteric ischemia can occur from primary mesenteric vascular insufficiency from venous thrombosis, arterial embolism, or thrombosis, or secondarily as a complication of low inflow (non-obstructive mesenteric ischemia) or aortic dissection (1–5). Regardless of its cause, acute mesenteric ischemia is a surgical emergency that entails complex general and vascular surgical management and is associated with significant morbidity and mortality (2, 5–8).

Mesenteric ischemia is the most frequent cause of peritonitis among critically ill patients and is a common indication for emergency bowel resection (9, 10). Among the elderly and critically ill, it is an independent risk factor for mortality (9, 11). Early mortality rates from mesenteric ischemia range from 20 to 80%, with survivors remaining at risk for significant long-term morbidity and late death (5–7, 12). However, the incidence and outcomes of acute mesenteric ischemia are poorly defined, in part due to limitations of institutional- and population-based studies to date (5–7, 12, 13). The purpose of this study was to evaluate the modern incidence and outcomes of acute mesenteric ischemia in a statewide database analysis.

## Materials and Methods

A retrospective analysis of mesenteric ischemia in the state of Maryland from 2009 to 2013 using the Health Services Cost Review Commission (HSCRC) database was performed. The HSCRC regulates hospital payment rates in Maryland and maintains a de-identified inpatient dataset that contains medical and billing data for all inpatient admissions to the state’s 51 hospitals (46 acute care, 3 psychiatric, and 2 specialty facilities) (14, 15). Data elements include demographics, hospital disposition, International Classification of Diseases (ICD-9) diagnosis and procedure codes, and All Patient Refined (APR) Diagnosis-Related Groups. Illness severity is categorized by the APR-severity of illness (SOI), an ordinal scale of minor, moderate, major, or extreme, reflecting the degrees of acute physiologic decompensation and organ dysfunction. Similarly, the Diagnosis-Related Group adjusted risk of death is classified by the APR-risk of mortality (ROM) using the same categorization (16, 17). The dataset was used under a Research Data Use Agreement approved by the HSCRC board, and the study was approved by the University of Maryland, Baltimore Institutional Review Board with a waiver for patient consent given the retrospective design and use of a de-identified dataset.

Patients at least 18 years old were included for analysis. Acute mesenteric ischemia was operationally defined as an ICD-9 code for acute intestinal ischemia (557.0 or 557.9) plus the need for abdominal surgery during the same admission (Table S1 in Supplementary Material) (18). The latter criteria were included to ensure clinically significant, and confirmed episodes of the disease were captured. Although potential readmissions could not be identified within the dataset, all admissions were assumed to be index events based on the natural history of mesenteric ischemia. Analyzed data included patient demographics, comorbidities, management, complications, and disposition. Statewide population data were obtained from the Maryland State Data Center (19), using census and intercensal estimates to determine the population at risk. Based on previous analysis of emergency general surgery in Maryland and resources available for acute surgical care, the state’s two Level I trauma centers were defined as tertiary referral centers for this study (16).

The primary outcome was inpatient mortality. Survivors and non-survivors were compared by chi-squared, Satterthwaite *t*, or Wilcoxon tests as appropriate. Variables associated with mortality on univariate analysis were assessed by multivariable logistic regression using automatic step-wise selection, and retained for a multivariable *P* < 0.01. For multivariate analyses, SOI was dichotomized as extreme or non-extreme, and bowel resection was categorized as small intestine or colon resection alone, or both, and compared to no bowel resection. Model calibration was assessed using the Hosmer–Lemeshow goodness of fit test. Data were analyzed using SAS 9.3 software (SAS Institute).

## Results

During the 5-year study period, there were 3,157,499 adults admitted to Maryland hospitals. A total of 2,255 adults had acute mesenteric ischemia as defined by ICD-9 diagnosis and need for abdominal surgery, representing 0.07% of inpatient admissions. Based on an average state population of 5.8 million during 2009–2013, the annual admission rate among Maryland adults was 10 per 100,000.

Patients with acute mesenteric ischemia were generally elderly, white, and female (Table 1). The majority of admissions (90%) were classified as urgent or emergent, with 81% of patients presenting *via* the emergency department. Only 17% of patients were admitted to tertiary hospitals. Compared to patients without mesenteric ischemia, the study group was older, more frequently Caucasian, and more likely to be admitted emergently and to a tertiary hospital. Specific mechanisms of ischemia were not discernable from the database, but among potential etiologies and contributing conditions, 198 (9%) patients had a hypercoagulable state, 37 (2%) had an arterial embolic event, and 12 (1%) had arterial dissection. Representing significant acute physiologic dysfunction and risk of death, 68% of patients with mesenteric ischemia had extreme APR-SOI, and 59% had extreme APR-ROM.

**Table 1:** **Patient demographics**.

Characteristic	No mesenteric ischemia	Mesenteric ischemia	*P*	Survivors	Non-survivors	*P*
Number	3,155,244	2,255		1,704	551	
Demographics, *n* (%)^a^						
Age, years ± SD	57 ± 20	67 ± 16	0.0001	66 ± 17	71 ± 15	<0.0001
Age >65 years	1,204,123 (38%)	1,321 (59%)	0.0001	939 (55%)	382 (69%)	<0.0001
Female	1,870,001 (59%)	1,321 (59%)	0.51	1,010 (59%)	311 (56%)	0.24
White	1,892,553 (60%)	1,617 (72%)	0.0001	1,231 (72%)	386 (70%)	0.32
Medicare insurance	1,358,289 (43%)	1,379 (61%)	0.0001	988 (58%)	391 (71%)	<0.0001
Admission, *n* (%)						
Emergent	2,280,960 (72%)	2,027 (90%)	0.0001	1,534 (90%)	493 (89%)	0.71
ED presentation	1,997,807 (63%)	1,824 (81%)	0.0001	1,402 (82%)	422 (77%)	0.003
Tertiary hospital	452,469 (14%)	385 (17%)	0.0001	241 (14%)	144 (26%)	<0.0001

*^a^Except as noted*.

*ED, emergency department*.

Chronic comorbidities and acute organ dysfunction were common among the study group (Table 2). On univariate analyses, hypercoaguability, cardiac dysrhythmia, chronic kidney disease, and increasing APR-SOI were associated with mesenteric ischemia; diabetes and hypertension were less common among patients with mesenteric ischemia. Heterogeneity within the study group limited multivariable analysis of factors associated with development of mesenteric ischemia. However, under multiple stratified models, tertiary hospital admission, extreme APR-SOI, hypercoagulability, and arterial embolic events were consistent independent risk factors for mesenteric ischemia.

**Table 2:** **Comorbidities and illness severity**.

Characteristic	No mesenteric ischemia	Mesenteric ischemia	*P*	Survivors	Non-survivors	*P*
Number	3,155,244	2,255		1,825	577	
Condition, *n* (%)						
Diabetes mellitus	786,591 (25%)	350 (16%)	<0.0001	279 (16%)	71 (13%)	<0.05
Hypertension	1,053,797 (33%)	607 (27%)	<0.0001	519 (30%)	88 (16%)	<0.0001
Hypercoagulable state	47,689 (2%)	198 (9%)	<0.0001	87 (5%)	111 (20%)	<0.0001
Ischemic heart disease	634,570 (20%)	405 (18%)	0.01	275 (16%)	130 (24%)	<0.0001
Cardiac dysrhythmia	625,989 (20%)	618 (27%)	<0.0001	423 (25%)	195 (35%)	<0.0001
PAD	130,649 (4%)	91 (4%)	0.80	65 (4%)	26 (5%)	0.35
Chronic comorbidities, *n* (%)						
COPD	345,330 (11%)	264 (12%)	0.25	185 (11%)	79 (14%)	0.03
Congestive heart failure	363,843 (12%)	259 (11%)	0.95	177 (10%)	82 (15%)	0.004
Chronic kidney disease	507,982 (16%)	861 (38%)	<0.0001	519 (30%)	342 (62%)	<0.0001
Severity of illness, *n* (%)			<0.0001			<0.0001
Minor	562,464 (18%)	5 (< 1%)		5 (< 1%)	0	
Moderate	1,208,321 (38%)	181 (8%)		181 (11%)	0	
Major	1,032,943 (33%)	540 (24%)		522 (31%)	18 (3%)	
Extreme	351,516 (11%)	1529 (68%)		996 (58%)	533 (97%)	
Risk of mortality, *n* (%)			<0.0001			<0.0001
Minor	1,526,896 (48%)	81 (3%)		81 (5%)	0	
Moderate	843,060 (27%)	245 (10%)		245 (14%)	0	
Major	550,003 (17%)	589 (25%)		550 (32%)	39 (7%)	
Extreme	235,285 (7%)	1,340 (57%)		828 (49%)	512 (93%)	

*PAD, peripheral arterial disease; COPD, chronic obstructive pulmonary disease*.

Small intestine involvement was the most common form of mesenteric ischemia based on procedure codes for bowel resections (38%; Table 3). Colonic involvement occurred in 27%, while 21% had both small and large bowel ischemia. However, 172 (7%) patients had no bowel resection, making their distribution unclear. Vascular intervention in this cohort was infrequent and was more common among non-survivors (7 versus 4%, *P* < 0.005). Patients who died required more intensive critical care management, including higher rates of mechanical ventilation, dialysis, and blood product transfusion.

**Table 3:** **Patient management**.

Characteristic	Mesenteric ischemia	Survivors	Non-survivors	*P*
Number	2,255	1,704	551	
Gastrointestinal surgery, *n* (%)				
No bowel resected	172 (7%)	70 (4%)	102 (19%)	<0.0001
Small bowel resection	920 (38%)	781 (46%)	139 (25%)	0.005
Large bowel resection	651 (27%)	518 (30%)	133 (24%)	<0.0001
Small and large bowel resections	512 (21%)	335 (20%)	177 (32%)	<0.0001
Vascular intervention, *n* (%)	99 (4%)	62 (4%)	37 (7%)	0.002
Open	11 (< 1%)	7 (< 1%)	4 (1%)	0.48
Endovascular	88 (4%)	55 (3%)	33 (6%)	0.004
Critical care, *n* (%)				
Mechanical ventilation	897 (37%)	494 (29%)	403 (73%)	<0.0001
Parenteral nutrition	774 (32%)	594 (35%)	180 (33%)	0.35
Dialysis	181 (8%)	90 (5%)	91 (17%)	<0.0001
Transfusion	1,031 (43%)	711 (42%)	320 (58%)	<0.0001

The overall mortality in the study group was 24%. On univariate analyses, increasing age, admission to a tertiary hospital, hypercoagulability, cardiac disease, renal insufficiency, illness severity, mechanical ventilation, dialysis, and transfusions were associated with mortality (Tables 1–3). Patients who died were more likely to require a combination of small and large bowel resection, or to undergo no resection at all. Age, illness severity, tertiary hospital admission, hypercoagulability, renal insufficiency, dysrhythmia, and respiratory failure were independent risk factors for mortality (Table 4). Compared to no bowel resection, resection of the small and/or large bowel was associated with similarly reduced mortality. Among survivors, 58% were discharged to home, 24% to a nursing facility, 9% to a rehabilitation center, and 6% to another acute hospital.

**Table 4:** **Multivariable analysis of risk factors for mortality**.

Variable	Odds ratio (95% CI)	*P*
Age > 65 years	1.8 (1.4–2.3)	<0.0001
Extreme severity of illness	2.8 (1.5–5.3)	<0.0001
Tertiary hospital admission	1.9 (1.4–2.5)	<0.0001
Hypercoagulability	2.6 (1.8–3.7)	<0.0001
Chronic kidney disease	1.8 (1.4–2.3)	<0.0001
Cardiac dysrhythmia	1.5 (1.1–1.9)	0.003
Mechanical ventilation	2.9 (2.3–3.8)	<0.0001
Bowel resection		
None (reference)	1.0	
Small intestine	0.13 (0.09–0.21)	<0.0001
Large intestine	0.15 (0.10–0.23)	<0.0001
Small + large intestine	0.19 (0.12–0.30)	<0.0001

*Hosmer–Lemeshow goodness of fit: χ^2^ = 8.9, *P* = 0.35*.

*c-Statistic = 0.85*.

## Discussion

Admission for mesenteric ischemia occurs in approximately 10/100,000 adults and in 1/1,000 inpatient admissions in Maryland. Though relatively uncommon, the disease complexity, illness severity, frequent requirement for operative intervention, and high associated mortality make it a significant burden on health care resources in Maryland. Findings in this study indicate that illness severity is the leading risk factor for mortality. The infrequency of traditional mechanisms of disease and low rate of vascular interventions suggest a high prevalence of low-flow mesenteric ischemia or hemodynamically occult mesenteric vascular disease. Definition of additional risk factors, including age, hypercoagulability, and comorbidities may help identify high-risk subgroups that may benefit physiologic optimization or possible intervention to prevent onset of mesenteric ischemia.

Few studies have assessed the epidemiology of mesenteric ischemia. In an analysis of the National Inpatient Sample by Beaulieu et al., 0.06% of patients had mesenteric ischemia (13). Based on extensive autopsy studies in Malmö, Sweden, Acosta estimated an incidence of 13/100,000 (3). While limitations of these studies may hinder their applicability and differences in methodology limit direct comparison between studies, these findings are similar to the 0.07% inpatient rate and statewide admission rate of 10/100,000 in the current study. Given the detail of this analysis and correlation with state population data, the study provides critical further insight into the epidemiology of mesenteric ischemia.

The complexity and heterogeneous pathogenesis of mesenteric ischemia has so far made it difficult to define specific risk factors for disease onset and mortality (2, 5, 20). This analysis demonstrated that advanced age, admission to a tertiary hospital, critical illness, hypercoagulability, chronic kidney disease, and dysrhythmia are associated with developing and dying from mesenteric ischemia. We were unable to evaluate specific etiologies of acute mesenteric ischemia in this dataset, but conditions traditionally associated with specific mechanisms such as embolism or hypercoagulability were relatively infrequent, suggesting a high prevalence of low-flow or hemodynamically occult mesenteric ischemia.

Consistent with a predominance of low-flow ischemia, illness severity and critical illness, as reflected by APR-SOI and need for mechanical ventilation, were the strongest risk factors for acute mesenteric ischemia onset and mortality. In the setting of critical illness, this could be attributable to true non-obstructive mesenteric ischemia or malperfusion superimposed upon otherwise occult chronic mesenteric vascular disease. Further study of risk factors specific to acute mesenteric ischemia in critically ill patients is warranted. Identification of high-risk patients, perhaps based on the presence of other risk factors or imaging characteristics, could help identify patients at increased risk for mesenteric ischemia and who may benefit from aggressive physiologic optimization and/or treatment of underlying vascular lesions.

Additional risk factors identified in this study, such as hypercoagulability and dysrhythmia, are consistent with traditional mechanisms of vascular occlusive mesenteric ischemia. Unfortunately, the nature and specific contribution of each are unclear. For example, these data do not indicate if hypercoagulability was potentially associated with mesenteric ischemia from venous versus arterial thrombosis. Similarly, dysrhythmia could contribute to vascular occlusive mesenteric ischemia from cardiogenic embolization or non-occlusive ischemia from low cardiac output.

Vascular interventions were infrequent in this study, which is consistent with a high prevalence of systemic malperfusion in the setting of critical illness. In this setting, optimal therapy entails optimization of systemic perfusion and resection of non-viable bowel, with limited role for vascular intervention. However, patients with hemodynamically occult chronic mesenteric arterial stenosis may be more susceptible to develop acute ischemia in the setting of systemic malperfusion. Further study is required to determine if such high risk patients would benefit from prophylactic intervention in the setting of critical illness. Under specific circumstances, such as embolic events with salvageable intestine, there is a clear role for vascular intervention, but this study indicates that these events are relatively infrequent.

Intuitively, bowel resection has a protective effect on mortality for patients with acute mesenteric ischemia. Patients in this study not undergoing bowel resection had significantly higher mortality than those who did. Presumably, patients who did not undergo bowel resection either had marginal bowel that was either viable or not appropriately resected, or non-survivable extensive involvement. While the distribution and extent of bowel involvement likely affects patient outcomes, different patterns of bowel resection were not independently associated with mortality. However, we were unable to account for the potential contribution of absolute length of resected bowel, number of resected segments, or final gastrointestinal continuity, which may affect short and long-term outcomes.

This study demonstrated a large burden of mesenteric ischemia managed at non-tertiary hospitals. Despite overall high mortality and disease complexity, the majority of patients were cared for at community facilities rather than transferred to tertiary centers. Further, tertiary hospitals had higher incidence and worse mortality even after adjusting for patient characteristics and illness severity. Given the growing regionalization of emergency general surgical care (21, 22), this finding warrants further exploration. The inter-facility transfer rate was, in the study, low but could represent a subgroup with disproportionately high mortality that could confound mortality at tertiary centers. Further, given the non-specific categorization of illness severity by the APR-SOI scale, the database may have failed to capture critical granular data that may further explain differences in risk and outcomes between community and tertiary hospitals, particularly for high-acuity critically ill patients who may be particularly susceptible to non-occlusive mesenteric ischemia.

The 24% mortality rate in this study is at the low end of the 20–80% short-term mortality rates reported in institutional series (2, 5–7, 12). Beaulieu et al. reported 36% mortality for mesenteric ischemia in the National Inpatient Sample but that analysis was limited to patients undergoing revascularization for mesenteric ischemia (13). As such, this comprehensive, population-based analysis may serve as a benchmark for early outcomes after mesenteric ischemia. While long-term outcomes data were not available in this dataset, the relatively high rate of home discharges among survivors suggests that meaningful recovery is achievable for many patients.

This study has important limitations. While the HSCRC database contains detailed patient level clinical data, the reliability of the results are limited by the use of an administrative database that was not designed for nuanced clinical analyses and depends on the quality of on-site data abstraction. Further, it lacks granular and time course data to give detailed insight into clinically important concerns such as the time between disease onset and intervention. While the admission rate in this study provides an approximation of incidence, determination of the true rate of mesenteric ischemia was confounded by two methodological factors. First, by using a de-identified database, we were unable to account for patient readmissions. Second, while operationally defining acute mesenteric ischemia by ICD-9 coding plus abdominal surgery may have improved the specificity of our study, we may have missed patients that died prior to hospitalization or were not surgical candidates. Finally, as a purely retrospective analysis, the ability to make definitive conclusions about the associations noted in the data is inherently limited.

## Conclusion

This study found a significant rate of mesenteric ischemia in a large, comprehensive statewide database and population analysis. These results demonstrated that critical illness is a leading risk factor for mesenteric ischemia onset and mortality. In our analysis, most patients are treated by bowel resection without vascular intervention, which suggests a significant burden of ­non-obstructive mesenteric ischemia or hemodynamically occult mesenteric vascular disease that is unmasked in the setting of critical illness. Additional risk factors may help identify patients at risk for this disease process. Mortality from mesenteric ischemia is high, but is significantly lower than suggested by prior studies that focused on acute vascular occlusive mesenteric ischemia. Further study is warranted to better define the interaction between patient and systematic risk factors.

## Author Contributions

RC conceived and planned the analysis, interpreted the data, and wrote the manuscript. DH planned the analysis, analyzed and interpreted the data, and wrote the manuscript. EK planned the analysis, analyzed the data, and reviewed and approved the manuscript. RT conceived the analysis, interpreted the data, and reviewed and approved the manuscript. JR interpreted the data and reviewed and approved the manuscript. HC analyzed the data, and reviewed and approved the manuscript. JD conceived and planned the analysis, interpreted the data, and reviewed and approved the manuscript.

## Conflict of Interest Statement

The authors declare that the research was conducted in the absence of any commercial or financial relationships that could be construed as a potential conflict of interest.

## Abbreviations

APR, all patient refined; HSCRC, health services cost review commission; ICD-9, International Classification of Diseases, Ninth Revision; ROM, risk of mortality; SOI, severity of illness.
