# Supplementary material for: A Statewide Analysis of the Incidence and Outcomes of Acute Mesenteric Ischemia in Maryland from 2009 to 2013
Source: Front Surg. 2016 Apr 14;3:22. doi: 10.3389/fsurg.2016.00022 (PMC4830818; doi:10.3389/fsurg.2016.00022)
Supplement: Supplementary file 1 [file Table_1.PDF]

Supplementary Table 1. Abdominal and vascular procedure codes.

ICD-9 Code	Procedure
	General abdominal
5411	Exploratory laparotomy
5412	Reopening of recent laparotomy
	Small intestine
4590	Intestinal anastomosis, not otherwise specified
4591	Small-to-small intestinal anastomosis
4561	Multiple segmental resection of small intestine
4562	Total removal of small intestine
4563	Ileostomy, not otherwise specified
4620	Temporary ileostomy
4623	Other permanent ileostomy
	Large intestine
4571	Open and other multiple segmental resection of large intestine
4572	Open and other cecectomy
4573	Open and other right hemicolectomy
4574	Open and other resection of transverse colon
4575	Open and other left hemicolectomy
4576	Open and other sigmoidectomy
4579	Other and unspecified partial excision of large intestine
4582	Open total intra-abdominal colectomy
4583	Other and unspecified total intra-abdominal colectomy
4610	Colostomy, not otherwise specified
4611	Temporary colostomy
4613	Permanent colostomy
	Diagnostic and endo-vascular
8842	Aortography
8847	Arteriography of other intra-abdominal arteries
0045	Insertion of one vascular stent
0046	Insertion of two vascular stents
0047	Insertion of three vascular stents
0048	Insertion of four or more vascular stents
3950	Angioplasty of other non-coronary vessel(s)
	Open vascular
3826	Resection of vessel with anastomosis, abdominal arteries
3926	Other intra-abdominal shunt or bypass

ICD-9, International Classification of Diseases version 9.
